# The Association of Obesity and Cardiorespiratory Fitness in Relation to Cognitive Flexibility: An Event-Related Potential Study

**DOI:** 10.3389/fnhum.2022.862801

**Published:** 2022-05-09

**Authors:** Tai-Fen Song, Chien-Heng Chu, Jui-Ti Nien, Ruei-Hong Li, Hsin-Yi Wang, Ai-Guo Chen, Yi-Chieh Chang, Kao-Teng Yang, Yu-Kai Chang

**Affiliations:** ^1^Department of Sport Performance, National Taiwan University of Sport, Taichung, Taiwan; ^2^Department of Physical Education and Sport Sciences, National Taiwan Normal University, Taipei, Taiwan; ^3^Graduate Institute of Athletics and Coaching Science, National Taiwan Sport University, Taoyuan, Taiwan; ^4^Center of Physical Education, Tzu Chi University, Hualien, Taiwan; ^5^College of Physical Education, Yangzhou University, Yangzhou, China; ^6^Physical Education Center, Chung Shan Medical University, Taichung, Taiwan; ^7^Institute for Research Excellence in Learning Science, National Taiwan Normal University, Taipei, Taiwan

**Keywords:** obesity, fitness, shifting, task-switching, event-related potentials, P3, N1

## Abstract

This study investigates an association between obesity and cardiorespiratory fitness concerning their potential effects on cognitive flexibility in young adults from behavioral and neuroelectrical perspectives. Eligible young adults (*N* = 140, 18–25 years) were assigned into one of four groups, according to their status of obesity (i.e., body mass index) and cardiorespiratory fitness levels (i.e., estimated maximal oxygen uptake), namely, normal weight with high cardiorespiratory fitness (NH), obese with high cardiorespiratory fitness (OH), normal weight with low cardiorespiratory fitness (NL), and obese with low cardiorespiratory fitness (OL). The task-switching test was utilized, and its induced endogenous (P3) and exogenous (N1) event-related potential components were recorded. Concerning behavioral indices, the NH demonstrated superior behavioral performance across global switching and local switching of the task-switching test compared to individuals with lower cardiorespiratory fitness and obesity (i.e., NL, OH, and OL). Additionally, the OH demonstrated better performance than the OL during the heterogeneous condition. For neuroelectrical indices, the NH had larger mean P3 amplitudes during global and local switching than the other three groups. A larger N1 amplitude was also observed in the NH during local switching than in the OH group. The findings suggest that cardiorespiratory fitness has beneficial effects on cognitive flexibility, attentional resource allocation, and sensory evaluation in young adults. Furthermore, our research provided novel evidence showing that cardiorespiratory fitness might potentially alleviate the adverse effects of obesity on cognitive flexibility in young adults.

## Introduction

Obesity has become a global pandemic, affecting approximately 650 million people worldwide, with 39% of adults older than 18 years considered overweight and 13% considered obese (World Health Organization, 2021). Obesity has not only been associated with a wide range of physiological complications, including metabolic syndrome, hypertension, type II diabetes, cardiovascular disease, and cancer (Marks et al., 2011; Kaaks and Kuhn, 2014; Suriagandhi and Nachiappan, 2022), but has also been correlated with a higher prevalence of mental health issues, such as emotional distress, anxiety, depression, and stress (Lavallee et al., 2021), as well as with a poorer health-related quality of life (Deckers et al., 2017; Lopez-Garcia et al., 2017).

Alternation in various aspects of cognitive function, including cognitive flexibility, has also been reported in the obese population (Yang et al., 2018). Cognitive flexibility refers to the ability to consciously switch between tasks sets or mental operations in response to task demands (Miyake et al., 2000; Diamond, 2013). Using various cognitive tasks tapping cognitive flexibility, researchers reported lower behavioral performance among individuals with obesity (Boeka and Lokken, 2008; Perpiñá et al., 2017; Testa et al., 2021), indicating greater cognitive flexibility deficits in individuals with obesity compared to those with normal weight. Notably, given the links between cognitive flexibility deficits and eating disorder attitudes (Edwards et al., 2018), cognitive flexibility deficits might incapacitate individuals with obesity from modifying existing behavioral patterns to lose weight or maintain healthy eating behaviors effectively.

Several potential mechanisms might cause the detrimental effects of obesity on cognitive function. For instance, obesity-induced low-grade inflammation (Lasselin et al., 2016; Shields et al., 2017) and elevated insulin resistance (Kim and Feldman, 2015) might lead to neuroinflammation of the central nervous system (Guillemot-Legris and Muccioli, 2017). Additionally, obesity has been associated with reduced cortical gray matters in the orbitofrontal cortex, inferior frontal gyrus, hippocampus, and anterior cingulate cortex (Raji et al., 2010; Herrmann et al., 2019; Chen et al., 2020), reduced cortical activities in the orbitofrontal cortex and medial prefrontal cortices (Tuulari et al., 2015; Zhang et al., 2015), as well as the altered bilateral orbitofrontal cortex and interhemispheric functional connectivity (Zhao et al., 2021; Song et al., 2022). The evidence suggests the negative linkages between obesity, structural and functional brain alternation, and cognitive function.

In addition to the overt behavioral indices (e.g., response time and accuracy), a growing number of studies have utilized the neuroelectrical approach [e.g., event-related potential (ERP)] to explore the potential underlying neuronal mechanisms. ERPs reflect the electrical activity associated with a specific task event or response (Fabiani et al., 2007). P3 component, the late positive deflection following the stimulus onset, has been utilized as the index of endogenous cognitive processing involving stimulus classification and attentional resources allocation, with larger P3 amplitude reflecting larger attention resource allocation (Polich, 2007). Notably, P3 amplitudes have been linked to body weights, such that smaller P3 amplitudes have been observed during cognitive tasks tapping cognitive flexibility, attention, and inhibition (Babiloni et al., 2009; Edwards et al., 2018; Liu et al., 2022), among individuals with heavier body weights. However, Nijs et al. (2008, 2010a,2010b) conducted a series of food-related studies that showed no significant differences in P3 amplitudes across individuals with different body weights. These inconsistent findings may arise from the cognitive tasks utilized and the underlying cognitive processes assessed. In contrast to P3 components, research examining the early exogenous N1 component, which reflects the initial sensory extraction and involves the preparation–perception–action cycle (Vogel and Luck, 2000), has been scantily conducted (Carbine et al., 2018).

Cardiorespiratory fitness (CRF) has also been associated with cognitive function. In fact, meta-analytic and systematic reviews have suggested the positive relationships between CRF and cognitive function (Reis et al., 2013; Kramer and Colcombe, 2018). Using a neuroelectrical approach, researchers have reported the superior inhibition performance, larger N1, and P3 amplitudes during a modified Erickson flanker task in individuals with higher CRF (Hillman et al., 2009; Pontifex et al., 2011; Berchicci et al., 2015), suggesting the positive effects of CRF on early sensory extraction and late attentional allocation. Individuals with higher CRF also demonstrated higher accuracy rates on a task-switching test (Verstynen et al., 2012) and in the local-global task (Zhan et al., 2020), revealing the beneficial effects of the CRF on cognitive flexibility. However, most studies related to cognitive flexibility have focused on the P3 component among populations with different fitness levels. A paucity of research exists examining the effects of CRF on the N1 component. Notably, while encouraging findings have been reported from exercise literature, only two studies have examined the effects of CRF on executive function between young adults with normal weight and young adults with obesity (Song et al., 2016; Chi et al., 2021). For instance, using the cross-sectional design, Song et al. (2016) reported that individuals with obesity and lower CRF levels had the longest response time during the Stroop incongruent trials, suggesting their deficits in inhibition. Interestingly, the authors noted that, regardless of the status of obesity, participants with higher CRF had comparable Stroop performances (Song et al., 2016). Similar findings of CRF on inhibition were reported by Chi et al. (2021), in which participants with higher CRF, regardless of the status of their obesity, had superior inhibition performance during the Stop-signal test. However, Song et al. (2016) have a methodological deficiency, where peak amplitudes from midline but not the averaged mean amplitudes from the regions of interest (ROIs) were measured for subsequent neuroelectrical analyses. Moreover, whether findings from Song et al. (2016) and Chi et al. (2021) could extend to other aspects of executive function (e.g., cognitive flexibility) remains unknown.

Although evidence has revealed the separate effects of obesity and CRF on cognitive function and the interaction between CRF and weight status on inhibition performance, the influence of CRF on other aspects of cognitive function among individuals with different weight statuses has not been well-studied. Accordingly, this study attempts to advance the knowledge by comparing cognitive flexibility performance among young male adults with different statuses of obesity and CRF by assessing behavioral indices (i.e., response time and accuracy) and neuroelectrical indices (i.e., N1 and P3) during a task-switching test. We expected that individuals with normal weight and higher CRF would demonstrate superior behavioral and neuroelectrical indices in cognitive flexibility. Moreover, individuals with obesity and lower CRF would exhibit the worst performance in cognitive flexibility. Finally, the deficits in the cognitive flexibility observed in individuals with obesity might be influenced by their CRF levels.

## Materials and Methods

### Participants

In total, 160 young male adults were initially screened over the phone and provided with an oral description of the experimental details. Participants were assessed based on the following inclusion criteria, namely, (a) ages between 18 and 25 years; (b) either being normal weight [body mass index (BMI):18.5–24 kg/m^2^] or obese (BMI > 27 kg/m^2^); (c) right-handed; (d) no history of brain injury, intellectual disability, or neurological disorders; (e) no history of cardiovascular disease; (f) no known physical disability and able to exercise; and (g) having a normal or corrected-to-normal vision. Finally, participants needed to either have higher CRF [i.e., maximal oxygen consumption (VO_2max_) > 70th percentile] or lower CRF (i.e., VO_2max_ < 30th percentile) levels (American College of Sports Medicine, 2022).

The final eligible participants (*n* = 140) were categorized into four groups according to their weight status and CRF levels (*n* = 35 for each group), namely, NH (i.e., normal weight and high CRF), NL (i.e., normal weight and low CRF), OH (i.e., obesity and high CRF), and OL (i.e., obesity and low CRF) groups. All participants provided written informed consent in accordance with the Institutional Review Board of the National Taiwan University.

### Submaximal Cardiorespiratory Fitness Assessment

Participants’ CRF levels were assessed by the Young Men’s Christian Association submaximal exercise test protocol (Golding et al., 1989; Young Men’s Christian Association, 2000) performed on an electronically braking cycle ergometer (Ergoselect 100/200 Ergoline GmbH, Germany). This protocol consists of three or more consecutive 3-min workloads intended to raise the heart rates (HR) to between 110 bpm and 85% of the age-predicted maximum HR for two consecutive workloads. The initial workload began at 150 kpm.min^–1^ (25 W) at a pedaling rate of 50 rpm. Participants’ HR values were recorded during the last 15–30 s of the second and third minutes of each stage. The average of these two recorded HR values during the first stage was used to determine the loading for the subsequent workloads. Finally, each participant’s VO_2max_ (ml.kg^–1^ .min^–1^) was estimated using the extrapolation method based on the HRs recorded at the last two workloads. Participants whose VO_2max_ ≥ 48.0 ml.kg^–1^.min^–1^ were identified as high CRF, whereas those with VO_2max_ ≤ 41.9 ml.kg^–1^.min^–1^ were identified as low CRF (American College of Sports Medicine, 2022).

### Task-Switching Test

The task-switching test was utilized to assess cognitive flexibility via the Neuroscan Stim software (version 2.0; Neuro Inc., El Paso, TX, United States). Participants were instructed to complete six blocks of 64 trials, including two homogeneous (i.e., 1–2 blocks) and four heterogeneous (i.e., 3–6 blocks) blocks. The first homogeneous block was entitled “less than/greater than” and consisted of numeric digits in a solid rectangular box (i.e., task set A: AA). Participants were instructed to press the left and right buttons with the index fingers of each hand for numeric digits less and greater than 5, respectively. The second homogenous block was entitled “odd/even” and consisted of numeric digits inside a dashed rectangular box (i.e., task set B: BB). Participants were instructed to press the left and right buttons with index fingers of each hand if the numeric digit was odd and even, respectively. The 3–6 blocks were heterogeneous in which the two tasks sets (i.e., task set A and task set B) were presented in the order of AABBAABB. During heterogeneous blocks, participants were instructed to respond according to the types of task sets. Accordingly, 50% of the trials were switch trials in which the current and previous stimuli belonged to different task sets (i.e., AB or BA), and the other 50% of the trials were non-switch in which the current and previous stimuli belonged to the same task sets (i.e., AA or BB). Participants were instructed to respond as quickly and accurately as possible. The response accuracy and response time for the correct response were recorded for the following conditions, namely, (1) homogeneous condition (i.e., 1–2 blocks), (2) heterogeneous condition (i.e., 3–6 blocks), (3) switch condition (AB or BA in the heterogeneous condition), and (4) nons-witch condition (AA or BB in the heterogeneous condition).

### Electrophysiological Assessment

Electroencephalography (EEG) output was recorded from 32 scalp electrodes embedded in an elastic cap (Neuroscan Quick-cap, Neuroscan Inc., VA, United States) and two electrodes placed on the left and right mastoids. The electrodes were mounted according to the international 10–20 system, and electrical impedances for all electrodes were maintained below 5 kΩ. Vertical and horizontal electrooculographic (EOG) activities were recorded from additional electrodes placed above and below the left eye (VEOG) and on the outer canthi of the left and right eyes (HEOG). EEG data were continuously collected using the NuAmps2 amplifiers (Scan 4.0, Compumedics Neuroscan) at a sampling rate of 500 Hz and digitized with a 12-bit A/D converter. All electrodes were re-referred to the averaged right and left mastoid, with the AFZ electrode site as the ground.

Offline EEG data processing was low-pass filtered (20 Hz, 24 dB/Oct). VEOG and HEOG were corrected by the algorithm (Semlitsch et al., 1986), and baseline correction was conducted using a -200 to 0 ms pre-stimulus time interval. EEG data was then epoched from 200 ms pre- to 1,000 ms post-stimulus onset. Trials with incorrect responses or exceeding ±100 μV were excluded from further analysis. Based on the visual inspection of the grand-averaged ERP waveform, the mean amplitudes of each electrode were derived from time windows 50–150 and 300–550 ms for N1 and P3 components, respectively. Electrodes were selected to represent ROIs, including the frontal (F3, Fz, F4), central (C3, Cz, C4), and parietal (P3, Pz, P4) regions. The final ERP amplitudes for each ROI were the averaged mean amplitudes across the electrodes in each ROI. In addition to the grand-averaged ERP waveform, the scalp topographies for P3 components of the global switching and local switching within the time frame across the four groups (i.e., NH, NL, OH, and OL) have also been provided.

### Laboratory Procedure

Each participant visited the laboratory on two separate days. During the first visit, participants completed the informed consent and digit-span test for working memory capacity. Participants’ heights, weights, and body compositions (i.e., body composition assessed by Tanita BC-601 Body Composition Monitor) were measured, and their physical activity levels were assessed by the International Physical Activity Questionnaire (IPAQ). Prior to the CRF assessment, the Physical Activity Readiness Questionnaire (PAR-Q) was completed to ensure safety for the submaximal CRF assessment. If participants met the including criteria, they were enrolled in the study. On the second visit, participants were instructed to sit in a sound-attenuated room and were fitted with a 32-channel quick-cap (Neuroscan Inc., VA, United States) for EEG data recording throughout the task-switching test. Participants were given instructions for the task-switching test and practiced 20 trials prior to the formal task-switching test. Each session lasted approximately 60 min. The interval between the first and second visit to the laboratory was between 5 and 7 days.

### Statistical Analysis

Statistical analyses were performed using SPSS (version 21.0; IBM Corp., Armonk, NY, United States). A one-way analysis of variance (ANOVA) was conducted to compare the demographic factors among the four groups. Response time and response accuracy were separately analyzed using a 4 (group: NH, OH, NL, and OL) × 2 (global switching: homogeneous and heterogeneous) mixed-model ANOVA, as well as 4 (group) × 2 (local switching: switch vs. non-switch) mixed-model ANOVA. Additionally, mean amplitudes for P3 were analyzed using a 4 (group) × 2 (global switching) × 3 (region: frontal, central, and parietal) mixed-model ANOVA, as well as 4 (group) × 2 (local switching) × 3 (region) mixed-model ANOVA. A similar analytic procedure was applied for N1 amplitudes. The Greenhouse–Geisser correction to correct for violations of sphericity and the Student–Newman–Keuls *post-hoc* comparisons were conducted using Bonferroni corrected *t-*tests. All statistical analyses were performed with a significance level of *p* < 0.05.

## Results

### Participant Characteristics

The participant’s demographic details are presented in Table 1. The one-way ANOVA revealed no significant differences in age or height among the four groups. Significant differences in weight [*F*(3, 136) = 89.29, *p* < 0.001], BMI [*F*(3, 136) = 121.81, *p* < 0.001], body fat (%) [*F*(3, 136) = 122.28, *p* < 0.001], scores on the digit-span forward test [*F*(3, 136) = 5.21, *p* < 0.01] and backward test [*F*(3, 136) = 4.06, *p* < 0.05], IPAQ [*F*(3, 136) = 10.33, *p* < 0.001], and VO_2max_ [*F* (3, 136) = 222.91, *p* < 0.001] were observed among the four groups. The Student–Newman–Keuls *post-hoc* test revealed that the OH and OL groups had higher values for weight, BMI, and body fat than the NH and NL groups (*p* < 0.05). The NH and OH groups had higher IPAQ and VO_2max_ values than the NL and OL groups (*p* < 0.05). These findings suggested that the participants were appropriately assigned to the four groups. Differences in other indices are presented by Table 1.

**TABLE 1:** Participant demographics and cardiorespiratory fitness among four groups (means ± SD).

Variables	Higher CRF		Lower CRF	
	NH (35)	OH (35)	NL (35)	OL (35)
Age (years)	20.77 ± 1.66	20.71 ± 2.11	21.86 ± 1.79	21.37 ± 2.32
Height (cm)	173.29 ± 3.94	175.60 ± 6.34	174.60 ± 6.14	173.89 ± 5.95
**Weight**				
Weight (kg)	64.46 ± 4.88^a^	89.37 ± 10.51^b^	65.66 ± 6.68^a^	100.14 ± 17.69^c^
BMI (kg/m^2^)	21.46 ± 1.32^a^	28.93 ± 2.01^b^	21.54 ± 1.52^a^	33.09 ± 5.47^c^
Body fat (%)	13.60 ± 3.42^a^	23.18 ± 3.99^b^	15.75 ± 3.53^a^	30.07 ± 4.93^c^
**Digit span**				
Forward	15.03 ± 0.89^a^	14.03 ± 1.56^b^	14.66 ± 1.03	14.03 ± 1.51^b^
Backward	9.97 ± 3.12^a^	8.00 ± 2.68^b^	9.97 ± 2.80^a^	8.71 ± 2.86
IPAQ (METs)	4704.46 ± 3632.00^b^	9213.40 ± 7512.00^a^	3129.11 ± 3544.00^b^	3621.03 ± 4721.00^b^
VO_2max_ (ml/kg/min)	55.39 ± 4.96^a^	53.00 ± 3.84^a^	36.07 ± 3.58^b^	35.53 ± 4.40^b^

*^(a, b), (a, c), (b, c)^Significant differences between groups, p < 0.05.*

*CRF, cardiorespiratory fitness; IPAQ, International Physical Activity Questionnaire; NH, normal-weight and high cardiorespiratory fitness; NL, normal-weight and low cardiorespiratory fitness; OH, obesity and high cardiorespiratory fitness; OL, obesity and low cardiorespiratory fitness.*

### Behavioral Indices

#### Global Switching

The two-way ANOVA revealed a main effect of group [*F*(3, 136) = 10.56, *p* < 0.001, partial η^2^ = 0.19]. The Student–Newman–Keuls *post-hoc* test revealed a shorter response time for the NH group (556.98 ± 21.40 ms) compared with the OH group (645.81 ± 21.40 ms), the NL group (655.21 ± 21.40 ms), and the OL group (726.58 ± 21.40 ms, *p* < 0.05). In addition, a shorter response time was also observed for the OH group (645.81 ± 21.40 ms) compared with the OL group (726.58 ± 21.40 ms, *p* < 0.05). The analysis also revealed a main effect for global switching [*F*(1, 136) = 803.64, *p* < 0.001, partial η^2^ = 0.86], with a shorter average response time for the homogeneous (525.39 ± 8.70 ms) condition than for the heterogeneous condition (766.91 ± 13.77 ms, *p* < 0.001).

**FIGURE 1:**
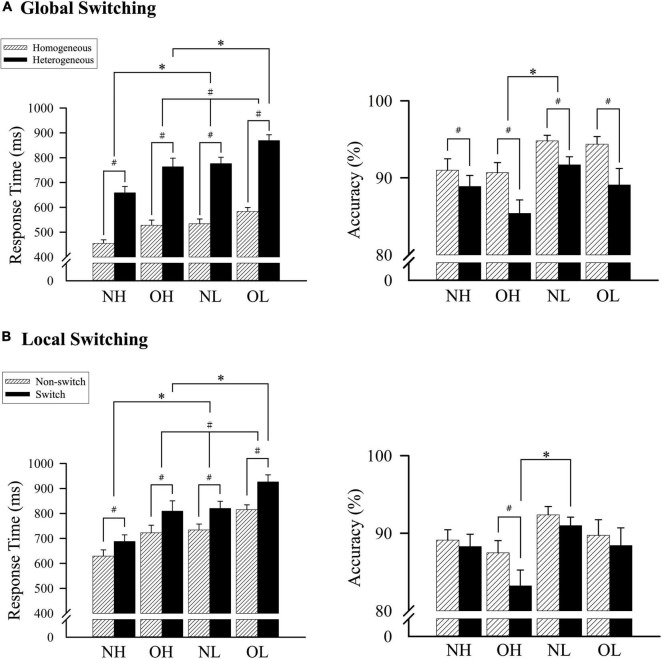
A comparison of the response time and accuracy among four groups according to **(A)** homogeneous and heterogeneous conditions and **(B)** nonswitch and switch conditions. NH, normal-weight and high cardiorespiratory fitness; NL, normal-weight and low cardiorespiratory fitness; OH, obesity and high cardiorespiratory fitness; OL, obesity and low cardiorespiratory fitness. ^#^Represents the significant difference between switch and nonswitch. *Represents the significant difference between groups.

A significant interaction was identified between group and global switching [*F*(3, 136) = 3.92, *p* < 0.05, partial η^2^ = 0.08]. Follow-up comparisons revealed a shorter response time for the NH group relative to OH, NL, and OL groups during the homogeneous condition (455.39 ± 17.40 ms vs. 528.03 ± 17.40 ms, 534.16 ± 17.40 ms, and 583.99 ± 17.40 ms, respectively, *p* < 0.05). Similar results were observed for the heterogeneous condition, in which the NH group had a shorter response time than the OH, NL, and OL groups (658.58 ± 27.53 ms vs. 763.60 ± 27.53 ms, 776.26 ± 27.53 ms, and 869.18 ± 27.53 ms, respectively, *p* < 0.05), and the OH group had a significantly shorter response time than the OL group (763.60 ± 27.53 ms vs. 869.18 ± 27.53 ms, *p* < 0.05). Additionally, all four groups had shorter response times for the homogeneous condition than for the heterogeneous condition (*p* < 0.001; Figure 1A).

Comparisons of response accuracy revealed a main effect of group [*F*(3, 136) = 2.99, *p* < 0.05, partial η^2^ = 0.06] and global switching [*F*(1, 136) = 49.92, *p* < 0.001, partial η^2^ = 0.27], but no significant interaction between group and global switching. Follow-up comparisons indicated that the NL group exhibited greater accuracy than the OH group (93.25% ± 1.31% vs. 88.03% ± 1.31%, *p* < 0.05), but no differences were observed between the NL, OL, and NH groups or between the OL, NH, and OH groups. The homogeneous condition resulted in higher accuracy than the heterogeneous condition (92.71% ± 0.58% vs. 88.76% ± 0.82%; *p* < 0.001; Figure 1A).

#### Local Switching

A two-way ANOVA revealed a main effect of group [*F*(3, 136) = 9.81, *p* < 0.001, partial η^2^ = 0.18], indicating that the NH group (658.60 ± 27.73 ms) exhibited a shorter response time than the OH group (766.08 ± 27.73 ms), the NL group (777.17 ± 27.73 ms), or the OL group (870.78 ± 27.73 ms, *p* < 0.05). The response time for the OH group was significantly shorter than that of the OL group (766.08 ± 27.73 ms vs. 870.78 ± 27.73 ms, *p* < 0.05). The analysis also revealed a main effect of local switching [*F*(1, 136) = 189.73, *p* < 0.001, partial η^2^ = 0.58], with a shorter average response time for the nonswitch condition (725.24 ± 12.44 ms) compared with the switch condition (811.07 ± 15.78 ms, *p* < 0.001).

An interaction effect was observed between group and local switching [*F*(3, 136) = 2.98, *p* < 0.05, partial η^2^ = 0.06]. Follow-up comparisons revealed a shorter response time for the NH group relative to the OH, NL, and OL groups during the nonswitch condition (629.22 ± 24.89 ms vs. 722.66 ± 24.89 ms, 734.02 ± 24.89 ms, and 815.07 ± 24.89 ms, respectively, *p* < 0.05), and the OH group had a shorter response time than the OL group (722.66 ± 24.89 ms vs. 815.07 ± 24.89 ms, *p* < 0.05). Similar results were observed for the switch condition, in which the NH group had a shorter response time than the OH, NL, and OL groups (687.98 ± 31.56 ms vs. 809.50 ± 31.56 ms, 820.32 ± 31.56 ms, and 926.48 ± 31.56 ms, respectively, *p* < 0.05), and the OH group had a significantly shorter response time than the OL group (809.50 ± 31.56 ms vs. 926.48 ± 31.56 ms, *p* < 0.05). Additionally, all four groups had shorter response times during the nonswitch condition than during the switch condition (*p* < 0.001; Figure 1B).

Comparisons of accuracy revealed no main effect of group, and only a main effect of local switching [*F*(1, 136) = 29.27, *p* < 0.001, partial η^2^ = 0.18] and an interaction effect between group and local switching [*F*(3, 136) = 4.68, *p* < 0.01, partial η^2^ = 0.09] were observed. Follow-up comparisons indicated that the nonswitch condition resulted in increased accuracy compared with the switch condition (89.68 ± 0.77% vs. 87.73 ± 0.90%, *p* < 0.001). Additionally, the OH group had significantly improved accuracy for the nonswitch condition than for the switch condition (*p* < 0.001), and the NL group had significantly improved accuracy compared with the OH group for the switch condition (90.99 ± 1.81% vs. 83.22 ± 1.81%; *p* < 0.05; Figure 1B).

### Event-Related Potential Indices

**FIGURE 2:**
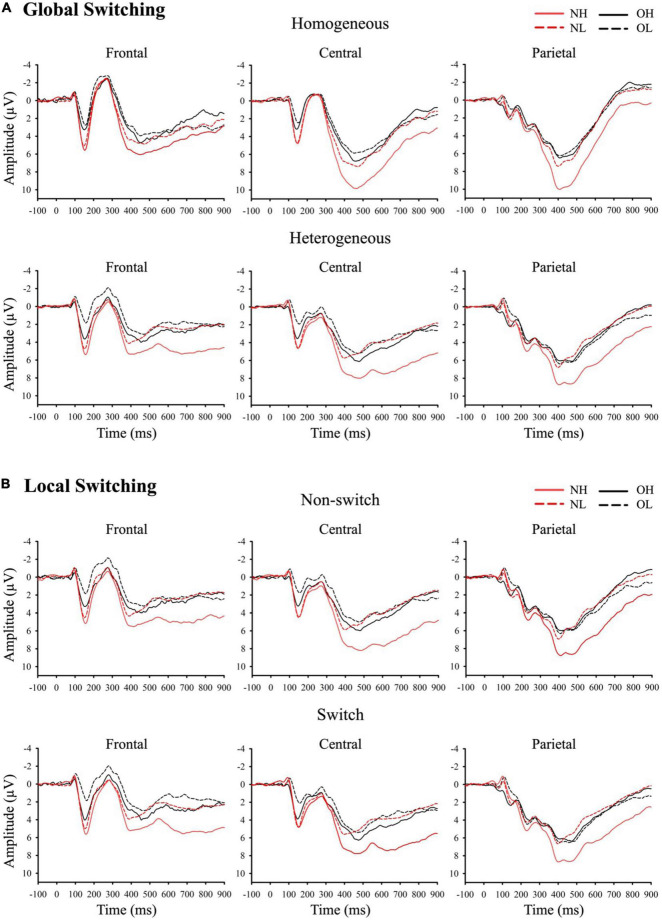
Grand-averaged ERP waveforms of **(A)** global switching and **(B)** local switching at frontal, central, and parietal regions. NH (i.e., normal-weight and high cardiorespiratory fitness), NL (i.e., normal-weight and low cardiorespiratory fitness), OH (i.e., obesity and high cardiorespiratory fitness), and OL (i.e., obesity and low cardiorespiratory fitness).

**FIGURE 3:**
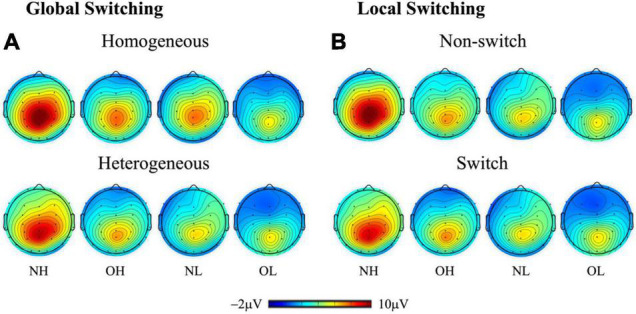
Topographic distribution (spectrum: blue to red) of the P3 amplitude (300–550 ms) for **(A)** global switching (i.e., homogeneous and heterogeneous conditions) and **(B)** local switching (i.e., nonswitch and switch conditions) across four groups: NH (i.e., normal-weight and high cardiorespiratory fitness), NL (i.e., normal-weight and low cardiorespiratory fitness), OH (i.e., obesity and high cardiorespiratory fitness), and OL (i.e., obesity and low cardiorespiratory fitness).

Figure 2 presents grand-averaged ERP waveforms of global and local switching. Figure 3 presents the topographic distribution of the P3 amplitudes of global and local switching.

#### Global Switching: P3 Amplitudes

The grand-averaged ERP waveforms for global switching were compared using a three-way ANOVA revealing a main effect of group [*F*(3, 136) = 7.95, *p* < 0.001, partial η^2^ = 0.15]. The Student–Newman–Keuls *post-hoc* test revealed a larger P3 amplitude for the NH group (6.88 ± 0.52 μV) than for the NL group (4.25 ± 0.52 μV), the OH groups (4.16 ± 0.52 μV), and the OL group (3.58 ± 0.52 μV, *p* < 0.01). No significant differences were observed between the NL, OH, or OL groups. The analysis also revealed a main effect of global switching [*F*(1, 136) = 12.78, *p* < 0.001, partial η^2^ = 0.09] and region [*F*(2, 272) = 114.59, *p* < 0.001, partial η^2^ = 0.46]. Follow-up comparison revealed a larger P3 amplitude for the homogeneous condition (5.07 ± 0.27 μV) compared with the heterogeneous condition (4.37 ± 0.29 μV, *p* < 0.001). In addition, the largest P3 amplitude was observed for the parietal region (5.74 ± 0.25 μV), followed by the central region (5.30 ± 0.30 μV), with the smallest P3 amplitude observed for the frontal region (3.12 ± 0.30 μV, *p* < 0.05).

An interaction between group and region was observed [*F*(6, 272) = 2.38, *p* < 0.05, partial η^2^ = 0.05]. Follow-up comparisons indicated that the frontal region had a smaller P3 amplitude than the central or parietal regions in the NH, OH, and NL groups (*p* < 0.001), but the P3 amplitude was smallest in the frontal region of the OL group, followed by the central region, with the largest P3 amplitude observed in the parietal region (*p* < 0.001). By contrast, the NH group had a larger P3 amplitude than the OL group in the frontal region, and the NH group had the largest P3 amplitudes relative to the other three groups in the central and parietal regions (*p* < 0.01).

Another interaction between global switching and region was observed [*F*(2, 272) = 22.75, *p* < 0.001, partial η^2^ = 0.14]. Follow-up comparisons indicated that the P3 amplitudes in the frontal region and central region were larger during homogeneous conditions than during heterogeneous condition (frontal: 3.53 ± 0.29 μV vs. 2.71 ± 0.34 μV; central: 5.84 ± 0.31 μV vs. 4.76 ± 0.33 μV, *p* < 0.001). Additionally, the frontal region had a smaller P3 amplitude than the central and parietal regions during the homogeneous condition (*p* < 0.001), whereas, during the heterogeneous condition, the frontal region had the smallest P3 amplitude, followed by the central region, with the largest P3 amplitude observed in the parietal region (*p* < 0.001).

#### Global Switching: N1 Amplitudes

The three-way ANOVA showed that no significant effect for the three-way interaction of group × global switching × region [*F*(6, 272) = 0.52, *p* > 0.05] or for any two-way interactions among group, global switching, or region. Additionally, no significant main effects were observed for either group [*F*(3, 136) = 2.79, *p* > .05, partial η^2^ = 0.06] or global switching [*F*(1, 136) = 0.04, *p* > 0.05, partial η^2^ = 0.01]. Only a main effect of region was observed [*F*(2, 272) = 17.93, *p* < 0.001, partial η^2^ = 0.12]. The N1 amplitude was largest in the frontal region (-0.27 ± 0.08 μV), followed by the central region (-0.12 ± 0.07 μV), with the smallest N1 amplitude observed for the parietal region (0.08 ± 0.08 μV, *p* < 0.01).

#### Local Switching: P3 Amplitudes

The three-way ANOVA revealed a main effect of group [*F*(3, 136) = 6.51, *p* < 0.001, partial η^2^ = 0.13], in which the NH group had the largest P3 amplitude (6.55 ± 0.58 μV) compared with the OH group (3.91 ± 0.58 μV), the NL group (3.69 ± 0.58 μV), and the OL group (3.32 ± 0.58 μV, *p* < 0.05), and no significant differences were observed between the OH, NL, and OL groups. The analysis also revealed a main effect of region [*F*(2, 272) = 119.03, *p* < 0.001, partial η^2^ = 0.47]. Follow-up comparison revealed that the largest P3 amplitude was observed for the parietal region (5.65 ± 0.26 μV), followed by the central region (4.75 ± 0.33 μV), with the smallest P3 amplitude observed for the frontal region (2.70 ± 0.34 μV, *p* < 0.001).

An interaction between group and region was observed [*F*(6, 272) = 2.33, *p* < 0.05, partial η^2^ = 0.05]. Follow-up comparisons indicated that the frontal region had a smaller P3 amplitude than the central and parietal regions for the NH, OH, and NL groups (*p* < 0.001), but the smallest P3 amplitude for the OL group was observed in the frontal region, followed by the central region, with the largest P3 amplitude observed in the parietal region (*p* < 0.001). By contrast, the NH group had a larger P3 amplitude than the OL group in the frontal region, and the NH group had the largest P3 amplitudes compared with the other three groups in the central and parietal regions (*p* < 0.01).

An interaction between local switching and region was observed [*F*(2, 272) = 11.77, *p* < 0.001, partial η^2^ = 0.08]. Follow-up comparisons indicated that the P3 amplitudes for both the switch and nonswitch conditions were the smallest in the frontal region, followed by the central region, with the largest P3 amplitudes observed in the parietal region (*p* < 0.001).

#### Local Switching: N1 Amplitudes

The three-way ANOVA revealed a main effect of group [*F*(3, 136) = 3.31, *p* < 0.05, partial η^2^ = 0.07], The NH group had a larger N1 amplitude (−0.44 ± 0.14 μV) than the OH group (0.15 ± 0.14 μV, *p* < 0.05). The analysis also revealed a main effect of region [*F*(2, 272) = 18.12, *p* < 0.001, partial η^2^ = 0.12]. Follow-up comparison revealed that the N1 amplitude was largest in the frontal region (−0.26 ± 0.08 μV), followed by the central region (−0.14 ± 0.07 μV), with the smallest N1 amplitude observed for the parietal region (0.08 ± 0.08 μV, *p* < 0.001). No significant effect was observed for the three-way interaction of group × local switching × region [*F*(6, 272) = 0.52, *p* > 0.05] or any two-way interactions among group, local switching, or region.

## Discussion

This study examined the influence of CRF on cognitive flexibility among individuals with different obesity statuses during the task-switching test. Our results supported our expectation, showing that young male adults with normal weight and higher CRF (i.e., NH) outperformed other young male adults (i.e., NL, OH, and OL) in both global switching and local switching of the task-switching test. Furthermore, the worst performance of all the task-switching conditions was found in the OL compared to the other three groups, which supported our second expectation, indicating the adverse effects of obesity on cognitive flexibility. Interestingly, individuals with obesity and higher CRF demonstrated better cognitive flexibility during the global switching than individuals with obesity and lower CRF. The neuroelectrical indices were closely emulated in the behavior results showing that the NH exhibited larger P3 amplitudes than the other three groups during global switching and local switching. Interestingly, the NH had a larger N1 amplitude during the local switching than the other three groups. These findings might suggest the importance of CRF and obesity status on cognitive flexibility in young adults.

We observed longer response times and decreased response accuracy during heterogeneous and switching conditions than during homogeneous and non-switching conditions, respectively, which is consistent with previous same-response task-switching protocol studies (Hillman et al., 2006; Kamijo and Takeda, 2010; Dai et al., 2013). The heterogeneous condition requires the maintenance and coordination of two alternating task sets, whereas the homogeneous condition only requires the ability to execute the one task set (Kray and Lindenberger, 2000). Additionally, responding to the heterogeneous blocks engages greater switching activity and requires more time to process a higher working memory load compared with responding to homogeneous blocks. The shifts between the switch and nonswitch trials within the heterogeneous block also explained the switching effect, which reflects the increase in task-set reconfigurations or switches recourse demands during task performance (Themanson et al., 2006).

The results of this study suggested the beneficial effects of CRF. The NH exhibited shorter response times during the homogeneous, nonswitch, and switch conditions, relative to the OH, NL, and OL, suggesting the beneficial effects of CRF on cognitive function. Higher response accuracy observed in the NH suggested no speed-accuracy trade-off effect. These findings of CRF-related benefits on cognitive function were in line with previous studies in the generally healthy population (Guiney and Machado, 2013), preadolescent children (Chu et al., 2019; Zhan et al., 2020), young adults (Song et al., 2016; Chi et al., 2021), late-middle-aged adults (Chu et al., 2016), and older adults (Chu et al., 2015). These benefits might result from altered connectivity between cortical regions and the prefrontal cortex (Chu et al., 2016; Peven et al., 2019). Our results further revealed significant differences in response times between the OH and the OL. Specifically, the OH demonstrated shorter response times in heterogeneous and local switching (switch/nonswitch) conditions compared to the OL, reflecting the meaningful role of CRF even among an obese population. This finding further supports the “fat-but-fit” paradigm reported in previous studies (Song et al., 2016; Chi et al., 2021), indicating that higher CRF might alleviate some of the adverse impacts of obesity. Collectively, the findings of this study extend the current knowledge of the benefits of CRF from inhibition (Song et al., 2016; Chi et al., 2021) to cognitive flexibility in the obese population.

Regarding neuroelectrical activities, our study observed a larger effect for the homogeneous than for the heterogeneous conditions on the P3 amplitude, which agrees with previous studies (Goffaux et al., 2006; Jost et al., 2008). The demand for working memory appears to be higher under heterogeneous conditions, resulting in the reduced availability of attentional resources. Similar effects were obtained for P3 amplitudes in response to local switching based on the comparison between nons-witching and switching conditions. Cue-locked P3 responses are thought to be associated with the updating of stimulus-response rules in working memory (Astle et al., 2008; Periáñez and Barceló, 2009). Task-switching studies often report parietal P3 responses (Gajewski and Falkenstein, 2012). The NH group exhibited the largest mean P3 amplitudes for both homogeneous and heterogeneous conditions during the task-switching test, which were significantly larger than the P3 amplitudes of the other three groups, with no significant difference observed among the OH, NL, and OL groups. The finding of the largest P3 amplitude indicates that the individuals with normal weight and higher CRF were capable of recruiting more attentional resources during the task-switching test. Previous studies have found that high-fitness individuals demonstrated larger P3 amplitudes than low-fitness individuals (Hillman et al., 2005; Pontifex et al., 2011). Interestingly, among individuals with obesity, improved CRF appeared to have a limited effect. Previous results have used the P3 amplitude to explore the relationship between obesity and executive function, reporting that obesity is associated with inferior inhibition (Tascilar et al., 2011; Kamijo et al., 2012). Our study further confirmed that obesity is associated with multiple aspects of cognitive domains, such as switching.

Except for the P3 component, which represents a late-stage endogenous component associated with allocating attentional resources, our study also examines the N1 component, an early-stage exogenous component that reflects the initial extraction of sensory information and sensory evaluation. We predict that CRF affects the N1 amplitude due to its effects to better identify the cognitive processes required to respond to stimuli. However, unlike the P3 component, our ERP data revealed a difference in the N1 amplitude under local switching conditions for the NH group compared with the OH group, suggesting the role of weight status in even high CRF during early sensory evaluation stages. Several studies have shown that the N1 amplitude is correlated with BMI (Key and Dykens, 2008), and a larger N1 amplitude was observed among high-fitness individuals compared with low-fitness individuals among both children and older adults (Chang et al., 2013; Berchicci et al., 2015), which supports our results.

Our strength is to examine the relationships among CRF, cognitive flexibility simultaneously, and obesity from both behavioral and neuroelectrical approaches, wherein concern for some limitations is required. First, given that the participants in this study were young male adults between 18 and 25 years, the results cannot be generalized for female adults and populations in varying age groups. Additionally, this study utilized a cross-sectional study design, preventing definitive conclusions regarding causation. Third, when using BMI to define obesity, some highly muscular but lean athletes can be classified as obese, and therefore, other obesity screening criteria may be used to measure body fat percentage indicators in the future. Another issue for possible consideration is the type of executive function. Studies have indicated that different cognitive tasks or food-related stimuli (Rutters et al., 2015) should be used in future studies associated with obesity. Lastly, uncontrolled factors (e.g., environment and nutrition) have been shown to influence neurocognitive performance in obesity (Duchesne et al., 2010; Khan et al., 2015); therefore, food intake measures in future studies should consider the potential effects of these relationships.

## Conclusion

Prior studies have examined the relationships between CRF and cognitive function in young adults, and this study replicated the benefits of CRF by showing that young male adults with normal weight and higher CRF demonstrated superior behavioral performance, enhanced sensory evaluation, and attentional resource allocation during the task-switching test compared to other young male adults. Furthermore, our results provided novel evidence showing that CRF might provide some protective effects reducing the adverse consequence of obesity on cognitive flexibility. The findings of this study not only support CRF as an important variable influencing cognitive flexibility but also inform the potential protective function against the adverse effects of obesity in young adults.

## Data Availability Statement

The raw data supporting the conclusions of this article will be made available by the authors, without undue reservation.

## Ethics Statement

The studies involving human participants were reviewed and approved by Institutional Review Board at National Taiwan University. The patients/participants provided their written informed consent to participate in this study.

## Author Contributions

T-FS, A-GC, Y-CC, K-TY, and Y-KC contributed to the conception of the work. Y-CC, K-TY, A-GC, and Y-KC contributed to the design of the study. T-FS, C-HC, K-TY, R-HL, and H-YW conducted the literature search, selection, and data extraction and analysis. T-FS, A-GC, C-HC, and J-TN wrote the first draft of the manuscript with support from J-TN, R-HL, H-YW, Y-CC, and Y-KC. All authors contributed to the manuscript revisions and agreed with final approval of the version.

## Conflict of Interest

The authors declare that the research was conducted in the absence of any commercial or financial relationships that could be construed as a potential conflict of interest.

## Publisher’s Note

All claims expressed in this article are solely those of the authors and do not necessarily represent those of their affiliated organizations, or those of the publisher, the editors and the reviewers. Any product that may be evaluated in this article, or claim that may be made by its manufacturer, is not guaranteed or endorsed by the publisher.
